# A Remarkable Surgical Operation

**Published:** 1891-02

**Authors:** 


A REMARKABLE SURGICAL OPERATION.—Professor Von Bergmann, of Berlin, is said to have lately conceived and carried out an operation which must be considered a marvelous tribute to the progress of modern surgery. Two patients were brought to him, one of whom was suffering under an injury which necessitated amputation of the thigh, and the other from a disease of the humerus, which called for excision of a part of that bone. The professor proceeded to operate upon the first of these patients, and he then removed the diseased portion of the bone from the arm of the second one, leaving necessarily a gap. This he actually filled with a portion of the healthy bone from the amputated leg, and a successful union was made. The second patient was by this clever operation endowed with a serviceable arm, instead of one which would probably have been useless.

